# Microglia preserve visual function in the aging retina by supporting retinal pigment epithelial health

**DOI:** 10.1186/s12979-023-00358-4

**Published:** 2023-10-14

**Authors:** Margarete M. Karg, May Moorefield, Emma Hoffmann, Hannah Philipose, Drenushe Krasniqi, Cindy Hoppe, Daisy Y. Shu, Shintaro Shirahama, Bruce R. Ksander, Magali Saint-Geniez

**Affiliations:** 1 Schepens Eye Research Institute of Mass Eye and Ear, 20 Staniford St, Boston, MA 02114 USA; 2 Department of Ophthalmology, Harvard Medical School, Boston, MA USA

**Keywords:** Microglia, PLX5622, Csf1r inhibitor, RPE, Retina, Visual function

## Abstract

**Background:**

Increased age is a risk factor for the development and progression of retinal diseases including age-related macular degeneration (AMD). Understanding the changes that occur in the eye due to aging is important in enhancing our understanding of AMD pathogenesis and the development of novel AMD therapies. Microglia, the resident brain and retinal immune cells are associated with both maintaining homeostasis and protection of neurons and loss of microglia homeostasis could be a significant player in age related neurodegeneration. One important characteristic of retinal aging is the migration of microglia from the inner to outer retina where they reside in the subretinal space (SRS) in contact with the retinal pigment epithelial (RPE) cells. The role of *aged* subretinal microglia is unknown. Here, we depleted microglia in aged C57/BL6 mice fed for 6 weeks with a chow containing PLX5622, a small molecule inhibitor of colony-stimulating factor-1 receptor (Csf1r) required for microglial survival.

**Results:**

The subretinal P2RY12 + microglia in aged mice displayed a highly amoeboid and activated morphology and were filled with autofluorescence droplets reminiscent of lipofuscin. TEM indicates that subretinal microglia actively phagocytize shed photoreceptor outer segments, one of the main functions of retinal pigmented epithelial cells. PLX5622 treatment depleted up to 90% of the retinal microglia and was associated with significant loss in visual function. Mice on the microglia depletion diet showed reduced contrast sensitivity and significantly lower electroretinogram for the c-wave, a measurement of RPE functionality, compared to age-matched controls. The loss of c-wave coincided with a loss of RPE cells and increased RPE swelling in the absence of microglia.

**Conclusions:**

We conclude that microglia *preserve* visual function in aged mice and support RPE cell function, by phagocytosing shed photoreceptor outer segments and lipids, therefore compensating for the known age-related decline of RPE phagocytosis.

**Supplementary Information:**

The online version contains supplementary material available at 10.1186/s12979-023-00358-4.

## Background

Aging is the most significant risk factor for age-related macular degeneration (AMD), which is a leading cause of blindness [1]. However, how age-related changes lead to an increased susceptibility to developing AMD is unclear. The primary cellular target in AMD is retinal pigment epithelial cells (RPE), which form a monolayer beneath the retina and play a central role in protecting and maintaining both the overlying photoreceptors and the choroidal vasculature. RPE cells are post-mitotic and perform specialized functions essential for retinal homeostasis, including maintenance of the blood-retina barrier, supplying photoreceptors with nutrients, eliminating waste products from photoreceptor cells, removing fluid from the subretinal space, and importantly, for functional vision, phagocytosis of photoreceptor-shed outer segments (POS) and visual pigment recycling [2, 3]. As RPE cells age, they undergo several changes, including a slow degeneration leading to an overall lower RPE cell density, an increase in cell size, accumulation of lipofuscin and other lipids, accumulation of basal deposits between the RPE cells and Bruch’s membrane, thickening of Bruch’s membrane, disorganization of basal infoldings, and microvilli atrophy [2, 4, 5]. These morphological and organizational changes correspond to decreased function of the remaining RPE cells, including reduced or delayed phagocytosis of POS [6, 7]. If we understand how aging RPE change and if we understand how these changes lead to an increased susceptibility to developing AMD, then this may lead to new therapeutic targets that decrease the risk of developing AMD in the elderly.

RPE cells have endogenous mechanisms that protect them from the stresses associated with increasing age, such as the multiple mitochondrial repair mechanisms that help RPE cells resist oxidative stress that is known to increase with age [8, 9]. In addition, RPE cells are also believed to be monitored externally by microglia for evidence of cellular stress and dysfunction. Even in young healthy mice there is evidence that microglia, located in the outer plexiform layer, extend long processes into the photoreceptor cell layer and subretinal space, presumably to monitor the environment for evidence of stress and/or cellular damage [10–12]. Microglia are known to execute multiple functions, such as maintaining the microenvironment, immunosurveillance, synaptic pruning, and regulating neurogenesis and axonal growth [13, 14]. In healthy adult retinas, the cell bodies of microglia are found largely in the inner retina, in the retinal ganglion cell layer (RGCL), inner plexiform layer (IPL), and outer plexiform layer (OPL) and are marked by a highly ramified morphology that coincides with a homeostatic functional state. Microglia are the most prominent resident immune cells of the central nervous system, and in the retina, they are the first to react to any cellular damage [15]. During homeostatic retinal conditions, microglia cell bodies remain in the inner layers of the retina, however, during retinal degeneration some migrate to the outer retina. Microglia also migrate to the outer retina and into the subretinal space, which is between the photoreceptors and RPE cells, during “normal” retinal aging [16–19] but whether these microglia either *delay* the age-related functional decline of the retina and RPE cells, or *accelerate* the aging process is unknown.

The study of microglia is currently a rapidly changing and quickly evolving field, due to the use of new technologies such as lineage tracing and single cell transcriptomics, which solved the previous problems associated with a lack of microglia-specific markers, resulting in an inability to identify, separate, and determine the function of microglia and infiltrating macrophages. Using these new techniques, Daniel Saban and coworkers [20] discovered that retinal microglia are heterogeneous and can be separated into two subpopulations in homeostatic adult retinas. Microglia within the inner plexiform layer are dependent upon IL-34 secreted from retinal ganglion cells and support cone bipolar cell function, thus helping to maintain normal visual signal processing. The second population of microglia reside within the outer plexiform layer and are IL-34 independent. Both of these microglia subpopulations migrate to the subretinal space during either acute light induced photoreceptor cell degeneration, or during chronic photoreceptor degeneration in Rho^P23H/WT^ mice with a knock-in mutation in rhodopsin. During these pathological conditions, microglia within the subretinal space are transcriptionally reprogrammed and *protect* RPE from morphological damage and promoted interdigitation of RPE microvilli with photoreceptor outer segments, which is essential for visual function. However, it is not yet known if these protective functions of microglia persist as the mice grow older, or whether the age-related decline of RPE is due, in part, to the loss of microglia activity.

While a study of aging retinal microglia has not yet been reported, Beth Stevens and coworkers [21] studied *brain* microglia in developing mice, during adulthood, following injury, and after the onset of old age at 18 months. Using single cell RNA-seq and in situ brain mapping, they identified nine different transcriptional microglia states that expressed unique gene signatures. Microglia were most heterogeneous during development and least heterogeneous in adult mouse brains. Brain microglia at the onset of old age possessed a small subpopulation of pro-inflammatory microglia that were equally distributed throughout the brain. Interestingly, brain injury produced microglia states that were similar to aging brain microglia [22]. While this study of aging brain microglia identified age-related transcriptional changes, how or if, these aging microglia affect brain functions has yet to be determined.

Our current study addresses the important functional connection between aging microglia and the aging outer retinal photoreceptors and RPE cells by eliminating microglia for six weeks at the onset of old age (18 months) and at the outer limits of the murine lifespan (36 months) and then examining the effects on visual function in mice with and without microglia by ERG a, b, and c waves and optomotor reflex (OMR). We found that microglia had no effect on photoreceptors as assessed by ERG measurements. However, even in extreme old age, microglia assist in protecting RPE survival and support RPE phagocytic activity by phagocytizing photoreceptor outer segments and lipids, resulting in a significant increase in ERG c wave, which is a measurement of RPE cell functionality and OMR responses. These data indicate that microglia interacting with RPE cells have protective effects on vision and specifically RPE cell function in very aged mice.

## Results

### Age-related increase of P2RY12 + cells in the neural retina

**Fig. 1:**
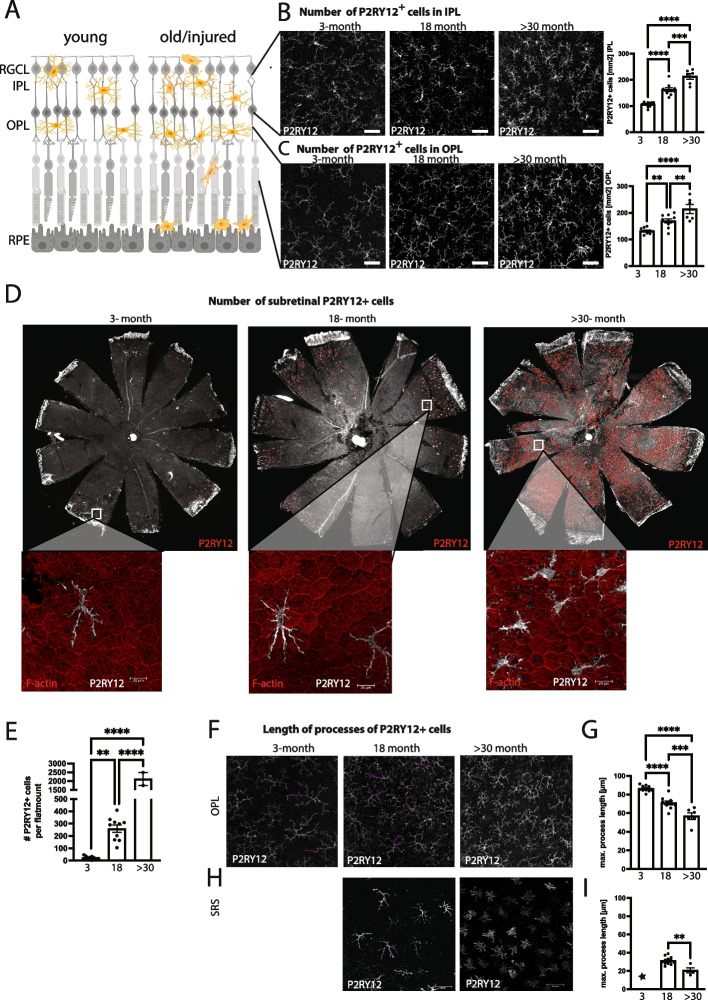
Age-related increase of microglia in inner plexiform layer (IPL), outer plexiform layer (OPL) and subretinal space (SRS). **A** Model of retinal cross sections showing changes in distribution and location of microglia between young/healthy and aged/injured retinas. **B** Representative images and number of P2RY12 + cells in IPL of 3-month, 18-month and > 30-month-old mice. **C** Representative max projected Z stacks images and number of P2RY12 + cells in OPL of 3-month, 18-month and > 30-month-old mice. **D** Representative RPE/choroidal flat mounts of 3-month, 18-month and > 30-month-old mice. P2RY12 + cells counted with ImageJ are labeled in red. High magnification zoom-outs stained with f-actin (red) and P2RY12 (white). **E** Total number of P2RY12 + cells on 3-month, 18-month and > 30-month RPE/choroid flat mounts. **F** Representative images of P2RY12 + cells in OPL of 3-month, 18-month and > 30-month-old mice. The 4 longest processes (purple) per image were traced and the process length measured using the NeuronJ plugin for ImageJ. **G** Maximal processes length of P2RY12 microglia in OPL at 3-month, 18-month and > 30 months. **H** Representative images of P2RY12 + cells in SRS of 18-month and > 30-month-old mice. The 4–5 longest processes (purple) per image and the process length was traced and measured using the NeuronJ plugin for imagej. **I** Maximal processes length of SRS P2RY12 microglia at 18-month and > 30 months. The number of P2RY12 cells were too rare at 3-months to quantify process length. *n* = 10 neural retinas per group, *n* = 10 RPE flat mounts for 3 months and 18 months, *n* = 2 RPE flat mounts for > 30 months, one-way anova with Tukey’s multiple comparison test. *P ≤ 0.05; **P ≤ 0.01; ****P ≤ 0.0001; ns, not significant. **B**, **F** and **H** scale is 50 µm, **D** scale is 20 µm

In healthy young retinas, microglia maintain retinal homeostasis and their location is limited to the retinal ganglion cell and inner plexiform layer (RGC / IPL) and outer plexiform layer (OPL) (Fig. 1A). P2RY12 is a specific microglia marker with significant higher expression in homeostatic microglia than macrophages, allowing discrimination between microglia and infiltrating blood derived macrophages [23, 24]. During aging, the number of P2RY12 + microglia significantly increased in the RGC/IPL and OPL (Fig. 1B and C) and microglia moved to the subretinal space (SRS). In young mice (3-months), very few P2RY12 + cells were found on the RPE apical side when imaged by flat-mounts. However, at the onset of old age (18-months) and very old mice (> 30-months), there was a significant age-dependent increase of SRS P2RY12 + cells (Fig. 1D and E). The increased number of microglia in aged mice coincided with significantly shorter maximal processes of P2RY12 + cells in the OPL (Fig. 1F and G). The subretinal P2RY12 + microglia also had significantly shorter maximal processes and displayed a highly amoeboid and activated morphology (Fig. 1H and I).

### Subretinal microglia support RPE by phagocytosing photoreceptor outer segments and lipids

**Fig. 2:**
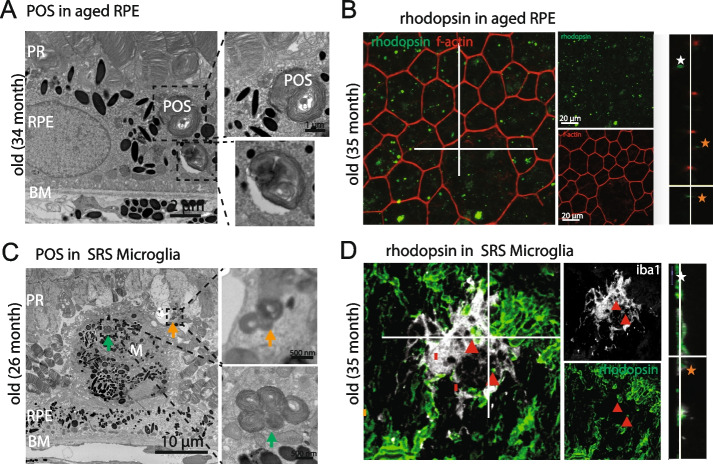
Phagocytized photoreceptor outer segments (POS) in aged RPE and subretinal microglia. **A**, **B** TEM and rhodopsin staining (rhodopsin—green, f-actin—red) revealed accumulation of undigested POS in aged RPE. **C**, **D** Phagocytosed POS are also observed inside subretinal microglia (green and orange arrow) by TEM and by rhodopsin staining (red arrows; rhodopsin—green, iba1—white). Colocalization of rhodopsin in orthogonal x–y cross sections inside the RPE and microglia(orange stars). Rhodopsin on top of the RPE or outside microglia (white stars). Images were taken from mice aged 26 – 35 months

RPE are responsible for phagocytizing shed photoreceptor outer segments (POS) to maintain the visual cycle, renew visual pigment, and facilitate visual signal transduction. A typical sign of age-related decline in RPE cell function is the accumulation of undigested POS that contain large amounts of rhodopsin [25–27]. In aged mice, as expected, we observed undigested POS in RPE cells via TEM (Fig. 2A) and, in RPE whole mounts, we observed accumulation of rhodopsin withing RPE via immunofluorescent staining with anti-rhodopsin antibodies (Fig. 2B). We also observed that microglia / macrophages within the SRS, which are on the apical side of RPE whole mounts, also contained POS via TEM (Fig. 2C) and, rhodopsin colocalized within Iba + cells via immunofluorescent staining (Fig. 2D).

**Fig. 3:**
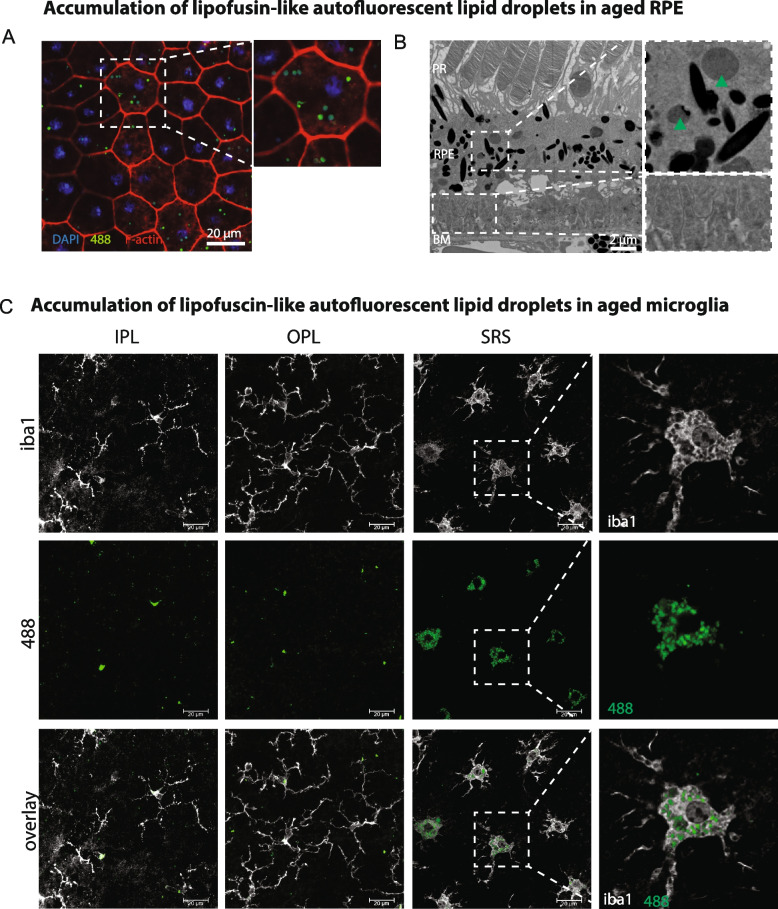
High density of lipofuscin-like lipid droplets in RPE and SRS microglia in aged mice. **A** Flat mount images of aged RPE labelled with F-actin (red) and DAPI (blue) showing accumulation of autofluorescent (488 nm wavelength) lipofuscin-like lipid droplets. **B** TEM imaging reveals numerous lipofuscin-like lipid droplets in aged RPE (green arrows) and accumulation of sub-RPE basal laminar deposits. **C** Aged microglia, in particular subretinal microglia accumulate high level of autofluorescent (488 nm wavelength) lipofuscin-like lipid droplets. Images were taken from mice aged 35 months

A second sign of the age-related decline of RPE cell function is the accumulation of lipofuscin-like autofluorescence lipid droplets. As expected, aging RPE showed accumulation of autofluorescent lipid droplets in RPE whole mounts (Fig. 3A) and via TEM (Fig. 3B). Autofluorescent lipid droplets were also found in microglia in aging retinas in the IPL and OPL but were observed at the highest density in microglia within the SRS (Fig. 3C). Together, these data indicate that microglia support the functions of aging RPE by migrating to the SRS and phagocytizing undigested POS and lipid droplets.

### Microglia depletion reduces visual acuity by optomotor response

**Fig. 4:**
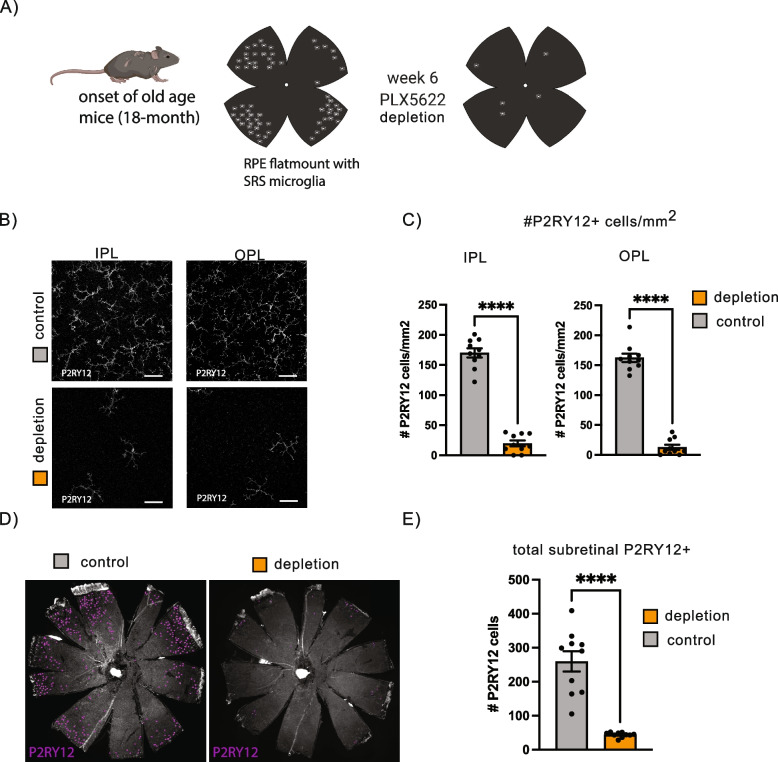
The PLX5622 diet effectively depleted P2RY12 microglia. **A** Study design summary showing 18-month mice were feed control or microglia depletion diet (PLX5622) for 6 weeks. **B** Representative high magnification images of IPL and OPL of and 18 months old control and PLX-treated mouse. **C** Quantification of P2RY12 + cells in IPL and OPL of control mice and mice on the depletion diet. **D** Representative RPE flat mounts of 18 months old mice on the control or microglia depletion diet. P2RY12 positive cells counted with ImageJ are labeled in purple. **E** Quantification of subretinal microglia per eye. *n* = 10 mice per group, unpaired t-test. **P ≤ 0.01; ****P ≤ 0.0001; ns, not significant. Scale = 50 µm

Microglia critically depend on colony-stimulating factor-1 (CSF1R) signaling [28–30] and therefore, we used the CSF1R antagonist PLX5622 to eliminate microglia in vivo. To confirm that PLX5622 treatment eliminated microglia in aging mice within the IPL and OPL, as well as, the microglia that migrate to the SRS, a group of mice at the onset of old age (18-months-old) received either the PLX5622 or a control diet for 6 weeks (Fig. 4A) after which RPE whole mounts were prepared, stained with P2RY12 antibodies, and the number of microglia quantified on confocal microscopic images. The PLX5622 diet significantly reduced the number of microglia in the IPL and OPL (Fig. 4B, C) and also reduced the number of microglia within the SRS (Fig. 4D, E), indicating this treatment is effective at eliminating microglia throughout the retina of elderly mice.

**Fig. 5:**
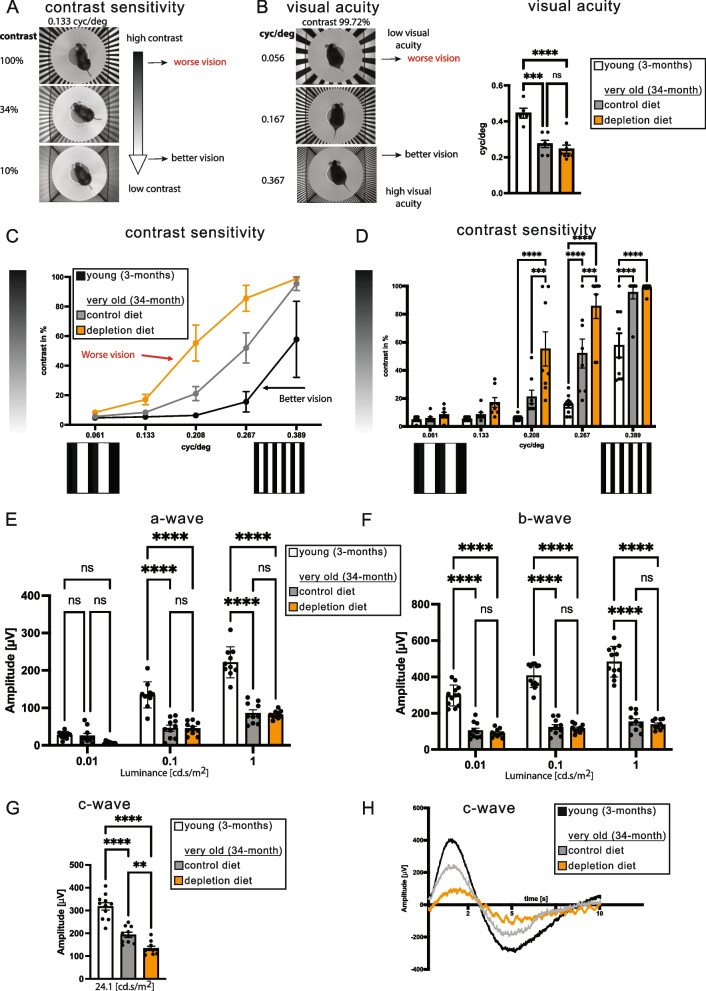
Microglial depletion reduces contrast sensitivity and ERG c-wave. **A** Contrast sensitivity function was measured using vertically oriented sinusoidal gratings of different contrast and spatial frequency (cycles per degree). Example of the OMR setup at 0.133cyc/deg to measure contrast sensitivity at full (99.73%), at 34% and at 10% contrast. Mice have worse vision when higher contrast is required to track the stripes. **B** Example of the OMR setup to measure visual acuity with increasing cycles per degree (0.056, 0.167 and 0.367) at maximum contrast. Significant reduction of visual acuity was measured between the young and old mice, but microglia depletion did not further affect the visual acuity measured at maximum contrast (99.73%). **C** Microglia-depleted mice showed reduced contrast sensitivity (higher contrast requirements corresponds to reduces contrast sensitivity and reduced vision) and **D** quantification revealed statistical significance at mid-range spatial frequencies. **E** Significant reduction of a-wave (**F**) and b-wave amplitudes was recorded between young and aged mice, but microglial depletion in older mice did not affect the a-wave and b-wave amplitudes. **G** Maximum c-wave amplitude was significantly reduced in aged mice compared to young and microglia depleted mice showed further reduction of c-wave amplitudes compared to the control mice. **H** Representative c-wave ERG comparing young (3-months) control and depletion diet. *n* = 8 mice per group, 2-way ANOVA, Šídák's multiple comparisons test. **P ≤ 0.01; ***P ≤ 0.001; ****P ≤ 0.0001; ns, not significant

We next wanted to use this PLX5622 treatment regime to determine the effect of depleting microglia on the visual function of aging mice. Three groups of mice at young adulthood (3-months-old), onset of old age (18-months-old), and at extreme old age (34-months-old) received a six-week treatment of either PLX5622 or a control diet and their visual function assessed using a variety of methods. Visual acuity was assessed using two types of optomotor responses (OMR) which measures the reflexive compensatory head movement exhibited by the mice in response to the rotation of black and white stripes. Visual acuity is measured by the response to rotating black and white stripes with decreasing spatial frequencies (Fig. 5B), while visual acuity using contrast sensitivity is measured by not only decreasing the spatial frequency, but also decreasing the contrast of the black lines (Fig. 5A). Importantly, it is believed that contrast sensitivity is currently the *most* sensitive method available to measure visual acuity [31]. Mice at the onset of old age (18-months-old) did not display any difference in visual acuity, including contrast sensitivity, between mice with and without microglia (Supplemental Fig. 1A-C). Mice at extreme old age (34-months-old), as expected showed a significant reduction in visual acuity as compared to young adult mice but displayed no significant difference in visual acuity in the presence or absence of microglia, as assessed by decreasing spatial frequency alone (Fig. 5B). By contrast, when contrast sensitivity was used to assess visual acuity in these mice, there was a significant *reduction* in acuity in the mice *without* microglia (Fig. 5C, D). Young mice displayed the highest acuity, which was reduced in the old mice treated with the control diet, but visual acuity worsened in old mice *without* microglia, indicating microglia were *protecting* the vision of old mice.

### Microglia depletion reduces visual function by electroretinogram c-wave responses

To validate our findings on changes in acuity using contrast sensitivity, we wished to confirm these results through a second assessment of visual function. Electroretinogram (ERG) assesses the electrical activity of the retina in response to a light stimulus. ERG c-waves measure the variation in the standing potential across Bruch’s membrane and is associated with the health of RPE cells [32]. ERG c-wave measurements displayed the highest activity in young adult mice, which significantly decreased in the extremely old mice (34-months-old) on the control diet, moreover, the c-waves of old mice without microglia were reduced even more (Fig. 5G). A representative c-wave pattern for individual mice is displayed in Fig. 5H and the responses of all mice are displayed in Fig. 5G. Therefore, via two separate measurements of retinal function, old mice without microglia displayed significantly reduced visual function, indicating a protective effect of microglia on RPE cells.

### Microglia depletion had no effect on ERG a and b waves

We performed additional ERG measurements to determine if the protective effects of microglia on the retina of extremely old mice were also extended to cells in the neural retina. ERG a-waves measure the electrical activity of photoreceptors, and b-waves reflects the health of the inner layers of the retina, including the ON bipolar cells and the Muller cells. As expected, young adult mice displayed the highest a- and b-wave responses (Fig. 5E, F) and there was a significant reduction in the response of old mice that received the control diet. However, there was no further changes in either a- or b-waves in old mice without microglia, indicating a specific RPE-related loss of function in the absence of microglia, whereas photoreceptor, ON bipolar cells and Muller cells were unaffected.

### Loss of RPE cells and increased RPE swelling in aged mice with depleted microglia

**Fig. 6:**
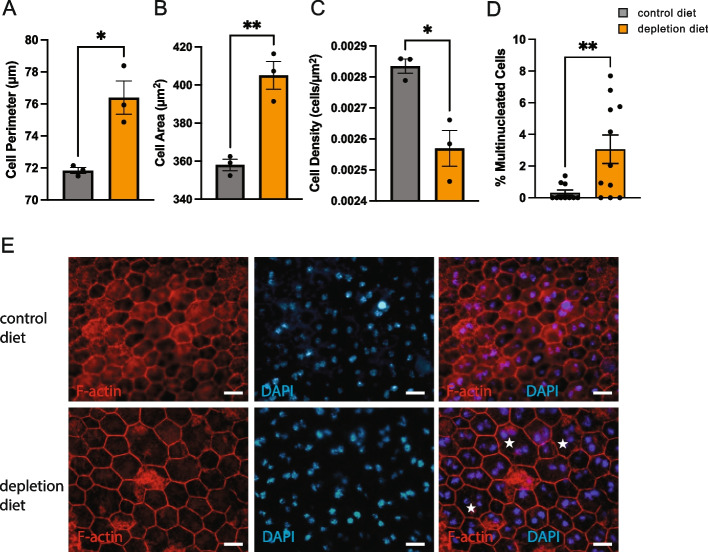
Microglia-depleted mice show morphological abnormalities in the RPE. Comparison of RPE **A** cell perimeter, **B** cell area, **C** cell density, and **D** percentage of multinucleated (≥ 3 nuclei per cell)of control versus microglia-depleted mice. **E** Representative images of RPE flat mounts acquired in the mid-periphery region and stained with F-actin (red) and DAPI (blue) show a regular hexagonal morphology with either single or binucleated cell in control compared to the enlarged and swollen RPE morphology with multinucleated cells (white stars) in microglia-depleted mice. Scale bar is 20 μm. *n* = 3 mice, unpaired t-test. *P ≤ 0.05; **P ≤ 0.01

RPE cells are post-mitotic cells with little to no renewal capabilities and, to ensure that the epithelial barrier is kept intact, they increase in size to compensate for lost cells. To assess the effects of microglia depletion on the morphology of RPE cells in the extremely old mice (34-month-old), RPE whole mounts were prepared from mice treated with either the PLX5622 or control diets. Images taken from the midperiphery were used to assess for RPE cell perimetry, cell area, cell density, and number of nuclei (Fig. 6A-D). Consistent with the protective effects of microglia on RPE cell function, in the *absence* of microglia, there was a significant *loss* of RPE cells (Fig. 6C), which coincided with an *increase* in the perimeter of the remaining RPE cells (Fig. 6A), an *increase* in the cell area (Fig. 6B), and in the percentage of multinucleated RPE cells (Fig. 6D). These morphological results are consistent with a protective effect of microglia on RPE in aging mice and indicate microglia promote RPE cell survival.

## Discussion

In this study we demonstrated that subretinal microglia in old mice protect from age-related vision loss by supporting RPE cell function. Microglia, as the resident immune cells of the CNS and the retina, execute multiple functions such as microenvironment maintenance, homeostasis by immunosurveillance, synaptic pruning, and regulation of neurogenesis and axonal growth [13, 14]. However, how these functions change during retinal aging is unclear, which is important to understanding the pathogenesis of age-related retinal diseases, such as AMD.

One of the challenges of aging research, even when using mice, is obtaining representative samples across the spectrum of aging, from onset until the end of lifespan limits. According to Jackson Laboratories “life phase equivalencies” between mice and humans [33], middle aged mice (10–14 months) are equivalent to middle aged humans (38 – 47 years), while onset of old age for mice (18–24 months) is equivalent to 56—69-year-old humans. During this phase, the percentage of surviving mice and humans declines to 80%. From this point to the upper limits of lifespan in mice (36 months) and humans (94 years), survival drops to the lower limits. In our study we examined mice at the onset of old age (18 months) and at the extreme limit of old age at 36 months.

We observed a significant age-related increase in microglia in the RGCL/IPL and OPL which is supported by previous studies in the retina and brains of mice, rats, and rhesus monkeys [17, 34, 35]. In addition, we also found an age-related increase of P2RY12 + microglia in the subretinal space that is adjacent to RPE cells which, in young healthy adult mice, is normally devoid of microglia [36].

However, it is known that during injury of the outer retina in young mice and in healthy older mice, microglia migrate into the SRS and acquire morphological features of activation, exhibiting large, round cell bodies and shorter processes [16–18, 20, 37]. Microglia in the outer retina of young mice during retinal degeneration was shown to protect RPE cells, but the function of microglia during aging is unclear.

Some investigators have suggested that during retinal aging there is increased para-inflammation characterized by microglial activation, subretinal migration, and blood-retinal barrier breakdown. At the retinal/choroidal interface para-inflammation is manifested by complement activation in Bruch’s membrane and RPE cells, and microglia accumulation in the subretinal space [38]. In aged mice, microglia have been observed migrating through the outer nuclear layer into the SRS [16, 17]. These data suggest that microglia in the outer retinas of aging mice may have a destructive pro-inflammatory effect.

Interestingly, we observed that the age-related increase in microglia coincided with reduced process lengths pointing towards a more activated state. Indeed, others have also shown that the total dendritic length was significantly reduced in the aged mice compared to the young [17]. Reduction of dendritic length and increase in cell body size has been correlated to an increased activated and inflammatory state of microglia, with negative effects on recovery and function [16, 39, 40]. Although studies suggest an inflammatory microglial phenotype in aged mice, RNA sequencing revealed that aged microglia display decreased expression of genes associated with endogenous ligand recognition and upregulated genes associated with microbe recognition and host defense [41]. Interestingly, aged microglia presented increased expression of genes related to neuroprotection and neurorestoration [41]. These findings indicate that despite the acquisition of pro-inflammatory features such as dendrite shortening and transition to an ameboid shape, these aged SRS microglial could still exert a beneficial and supportive function for RPE and/or photoreceptors.

RPE cell loss and slowing POS processing, resulting in the accumulation of undigested POS and lipids droplets are typical signs of the age-related decline in RPE cell function and may predispose the aging aye to AMD development [25–27]. We observed POS and autofluorescent lipid droplets also in the aged SRS microglia, indicating that they are performing similar tasks as the RPE cells. Like RPE cells, microglia are known to be highly phagocytic and have the capacity to remove dead cells and debris. Thus, microglia may migrate into the subretinal space as a compensatory mechanism for age-related RPE functional decline by supporting phagocytosis of debris/lipids and POS. Indeed, the absence of microglia led to the accumulation of dead photoreceptors and debris in the SRS [42]. The failure to clear debris, POS, and accumulated lipids could lead to cytotoxic effects, explaining the defects and reduced function of RPE cells we observed in the absence of microglia.

To study the function of the aged microglia in the retina, we fed aged mice with a chow containing PLX5622, a colony-stimulating factor-1 (CSF1R) inhibitor. Microglia are critically dependent on CSF1R signaling for their survival [43]. Csf1r-/- mice have a shortened lifespan, neurodevelopmental abnormalities and have highly reduced peripheral tissue-resident macrophage populations and brain-wide absence of microglia [44, 45], emphasizing the importance of CSF1R signaling for the survival of microglia. Grabert et al. [46] developed a transgenic mouse line with a reporter CSF1R protein expression. They showed that CSF1R expression is restricted to the myeloid cell lineage and is highly expressed in myeloid progenitors, monocytes, macrophages, and microglia but is significantly lower in granulocyte–macrophage progenitors and completely absent from granulocytes and lymphocytes [46]. Even though the expression of CSF1R is not limited to microglia, treatment with PLX5622, a CSFR1 antagonist, is highly effective at eliminating brain and retinal microglia in vivo [28, 29], with minimal effects on the number of immune cells in the spleen, bone marrow and peripheral tissues [47, 48].

As previously reported [49], we also observed an age-depended decrease in ERG a- and b-wave amplitudes, indicating a decline in the function of photoreceptors, bipolar, and Muller glial cells. Our results showed an additional age-related loss in c-wave amplitude, an indication of the loss of RPE cell function with age.

While the age-related decrease in a- and b-wave amplitudes did not decrease any further when microglia were depleted, there was a significant further reduction in the c-wave amplitude in PLX5622-fed old mice as compared with old mice feed with the control diet. These findings indicate that while there is an age-related decline in retinal function, microglia depletion did not further effect photoreceptor and secondary neuronal functions in the retina, whereas microglia depletion caused a further decline in age-related deficits in the RPE.

In retinal degeneration models in young mice, microglia in the SRS are not pathogenic, despite their activated morphology, but rather are involved in restricting disease progression [42]. Pathogenic conditions seem to trigger microglia relocation to the SRS and significant transcriptional reprogramming, characterized by reduced expression of homeostatic checkpoint genes and upregulation of injury-responsive genes, thought to enhance their protective role against RPE damage [20]. Similarly, aged microglia have been shown to undergo transcriptional, morphological and functional remodeling that is generally beneficial and essential for recovery, including upregulation of genes related to neuroprotection and neurorestoration [41, 50]. Since we showed that the absence of microglia in the SRS is directly linked to a loss of function of the RPE cells, it indicates that the translocation to the SRS and the transcriptional reprogramming of the aged microglia has a protective effect on RPE functionality and visual function.

## Conclusions

Overall, these data show that microglia play a beneficial role in protecting RPE during the age-related decline in visual function. Following microglia depletion, visual function in very old mice declined compared to the age-matched control group. This loss of visual function in microglia-depleted mice is associated with a significant reduction in RPE density and swelling of the remaining cells, indicative of degeneration and/or atrophy. We found that the subretinal microglia contained POS and high level of lipids, indicating that the SRS microglia offset some of the reduced phagocytic function of the aged RPE cells. In conclusion, microglia play an important role in *preserving* visual function in aged mice in particular SRS microglia by supporting RPE cell function.

## Materials and methods

### Mice

C57BL6 wild-type mice were housed under 12-h light/dark cycles (6:00/18:00), at an ambient temperature of 70–72 °F (21–22 °C) and 40–50% humidity. All animal procedures were reviewed and approved by the Institutional Animal Care and Use Committees (IACUCs) at Schepens Eye and Ear of Mass Eye and Ear according to appropriate animal welfare regulations.

### Mice anesthesia

Mice were anesthetized by intraperitoneal injection of a mixture of ketamine/xylazine (100–200 mg kg^−1^/20mgkg^−1^) supplemented by topical application of proparacaine to the ocular surface (0.5%; Bausch & Lomb). For quicker recovery, mice were injected intraperitoneally with yohimbine (2 mg/kg) to counteract the anesthesia effects of xylazine, after the procedures.

### Depletion of microglia with the CSF1R inhibitor PLX5622

Microglia depletion was performed using a selective CSF1R inhibitor PLX5622 (Plexxikon Inc.), incorporated into rodent chow. The regular chow of mice was either replaced by the control chow (AIN-76) or chow containing 1,200 ppm of PLX5622 for 6 weeks. Geriatric mice were closely monitored by the attending veterinarian and euthanized or excluded from the study when significant physiological and/or behavioral issues were observed (e.g. for severe dermatitis, poor overall condition) during the study. No obvious behavioral or health problems were observed because of the PLX5622-supplemented diet.

### Visual acuity and contrast sensitivity

The visual acuity and contrast sensitivity of mice was measured using an automated optomotor reflex-based spatial frequency threshold test using the Optodrum (Striatech). Mice were placed on a pedestal in the center of an area formed by four computer monitors arranged in a quadrangle. The monitors displayed a moving vertical black and white sinusoidal grating pattern adjusted to mouse movement to ensure equal distance from the mouse to the pattern. The software captured the outline of the mice. Nose and tail pointers were used to assess tracking behavior of mice automatically. Tracking of both eyes were assessed within the same trial by changing the rotation of the displayed pattern. The tracking is recorded only when the mice are sitting still, and mice were tracked for a maximum of 20 s still-sitting-time. Tracks in the counter direction are subtracted from the total tracking and if the threshold surpasses the trial is considered positive. For visual acuity the contrast of 99.27% and rotation speed (12° s^−1^) was kept constant and the cycle per degree were adjusted in a preprogrammed staircase method. Contrast sensitivity was measured at five cycle per degree (0.061, 0.133, 0.208, 0.267 and 0.389). Two positive and three negative trials at the next higher cycle per degree are required to confirm the final outcome.

### Electroretinogram

The mice were dark adapted overnight and were kept under dim red light throughout the procedure on a built-in warming plate (CELERIS, Diagnosys, Lowell, MA) to maintain their body temperature at 37 °C. A-wave and b-wave amplitudes were measured at 0.01, 0.1 and 1 cd.s/m2. C-wave was measured at a flash intensity of 24.1 cd.s/m2. The analysis was performed using the CELERIS software (Diagnosys, Lowell, MA). The a-wave amplitude and implicit time were measured from the baseline to the trough of the first negative wave; the b-wave amplitude was measured from the trough of the a-wave to the peak of the highest positive wave; the c-wave amplitude was measured from the baseline to the maximum recorded peak.

### Immunofluorescence – RPE/choroidal and neuroretina flat mounts

Eyes were enucleated and fixed overnight in 4% PFA at 4 °C. Neural retina (NR) and RPE/choroidal flat mounts were prepared by removal of the anterior segment and separation of the two ocular tissues. Radial cuts from the periphery toward the center were made to flatten the layers. RPE/choroid and NR tissue were incubated in blocking buffer (1% BSA, 0.1% TritonX, 3% donkey serum in PBS) for 1–2 h. Then, the tissues were incubated for 3 days at 4 °C with the following primary antibodies in blocking buffer: Anti-Phalloidin-594 (Invitrogen), Anti-Iba1 antibody (EPR16588, Abcam), Anti-Rhodopsin (Rho 4D2, Abcam), Anti-P2RY12 (AnaSpec, AS-50043A). To distinguish microglia from infiltrating macrophages, retinae from old mice were stained with P2RY12, a microglia-specific marker. After washing with PBS, the tissues were incubated with secondary antibodies (Donkey Anti-Rabbit IgG Alexa Fluor 647 (GR3408408-1, Abcam), Donkey pAb to rabbit IgG (DyLight 650 (GR3349963-2, Abcam), Alexa Fluor 647 Goat anti-Rabbit IgG (A21245, Invitrogen), Alexa Fluor 488 Goat Anti-Mouse IgG (A11001, Invitrogen)) and cell nuclei were stained with DAPI (D9542, sigma) (10 µg/ml for RPE/choroid and 1 µg/ml for NR tissue). The slides were mounted with mounting medium (Polyvinyl alcohol (PVA) with DABCO).

### Microglia counting and morphology analysis

A total of four image stacks of each retinal flat mount were acquired with the confocal microscope (SP8, Leica microsystems). ImageJ was used to separate total retinal stacks into RGCL/IPL and OPL stacks. Max. projections for each layer were created and total number of P2RY12 + microglia were counted using the ImageJ cell counter plugin. The average lengths of the four/five longest microglia processes per image were used as a morphological marker for the activation status of the microglia and were measured using ImageJ NeuroJ plugin. Cell counting and process length measurements were performed by masked individuals.

### RPE morphology

To assess RPE morphology and nuclei number, high magnification images were taken of RPE/choroid flat mounts with an Axioscope microscope (Carl Zeiss Microscopy) or a confocal microscope (SP8, Leica microsystems) whereby RPE cell borders were defined by f-actin using anti-Phalloidin-594 (Invitrogen) and nuclei by DAPI (D9542, Sigma) staining. Images were given numerical codes and analyzed by one grader masked to the diet group. The grader excluded images with > 20% of cells out of focus or otherwise difficult to assess. For RPE morphology assessments all visible cells with an entire and defined cell border were manually traced using ImageJ. A minimum of 20 cells were analyzed per image, and cell area (μm^2) and cell perimeter (μm) were recorded. Cell density was calculated using the formula (# of cells)/(sum of cell areas in μm^2), and form factor was calculated using the formula (4π * cell area)/(cell perimeter^2). To analyze the RPE nuclei number, a minimum of 20 cells were analyzed per image and nuclei per RPE cell were counted using the ImageJ cell counter plugin. RPE cells with 3 or more nuclei were defined as multinucleated cells. The percentage of counted multinucleated cells per image was calculated.

### TEM analysis

Mice were anesthetized with ketamine/xylazine (100 mg kg^−1^ and 20 mg kg^−1^). Live animals were perfused via the aorta with 10 ml of sodium cacodylate buffer (0.1 M, pH 7.4) followed by 10 ml of 1⁄2 Karnovsky’s fixative in 0.1 M sodium cacodylate buffer (Electron Microscopy Sciences). Eyes were enucleated and the anterior segment removed. The eyecups were dehydrated and embedded in tEPON-812 epoxy resin. Semithin Sects. (1 μm) were stained with 1% toluidine blue in 1% sodium tetraborate aqueous solution for light microscopy. Ultrathin Sects. (80 nm) were cut from each sample block using a Leica EM UC7 ultramicrotome (Leica Microsystems) and stained with 2.5% aqueous gadolinium triacetate hydrate and Sato’s lead citrate stains using a modified Hiraoka grid staining system [51]. Grids were imaged using an FEI Tecnai G2 Spirit transmission electron microscope (FEI Company) at 80 kV interfaced with an AMT XR41 digital CCD camera (Advanced Microscopy Techniques) for digital TIFF file image acquisition. TEM imaging of retina samples was assessed, and digital images were captured at 2 k × 2 k pixel, 16-bit resolution.

### Statistical analysis and figure preparation

Statistical analyses were performed with GraphPad Prism 9, using *t*-tests, one-way or two-way ANOVA. All statistical tests performed are indicated in the figure legends. The data are presented as mean ± SEM unless otherwise indicated.

### Supplementary Information

**Additional file 1: Supplemental Fig. 1.** Microglia depletion in 18 months mice but does not affect visual function. Microglia-depleted middle-aged mice (18-months) showed no difference in (**A**) visual acuity, (**B**) contrast sensitivity, and (**C**) ERG (a, b and c – waves).

## Data Availability

All data generated or analysed during this study are included in this published article and its supplementary information files.
