# Correction: Microglia preserve visual function in the aging retina by supporting retinal pigment epithelial health

**DOI:** 10.1186/s12979-023-00388-y

**Published:** 2023-11-11

**Authors:** Margarete M. Karg, May Moorefield, Emma Hoffmann, Hannah Philipose, Drenushe Krasniqi, Cindy Hoppe, Daisy Y. Shu, Shintaro Shirahama, Bruce R. Ksander, Magali Saint-Geniez

**Affiliations:** 1 Schepens Eye Research Institute of Mass Eye and Ear, 20 Staniford St, Boston, MA 02114 USA; 2 Department of Ophthalmology, Harvard Medical School, Boston, MA USA

**Correction: Immun Ageing 20, 53 (2023)**

10.1186/s12979-023-00358-4

Following publication of the original article [1], the authors reported an error in the article title. The word “loss” should be removed. The correct article title should be “Microglia preserve visual function in the aging retina by supporting retinal pigment epithelial health”.

The original article [1] has been updated.
