# Supplementary figures and images for: Microglia preserve visual function in the aging retina by supporting retinal pigment epithelial health
Source: Immun Ageing. 2023 Oct 14;20:53. doi: 10.1186/s12979-023-00358-4 (PMC10576380; doi:10.1186/s12979-023-00358-4)

# Supplemental Figure 1: Microglia depletion in 18 months mice but does not affect visual function.

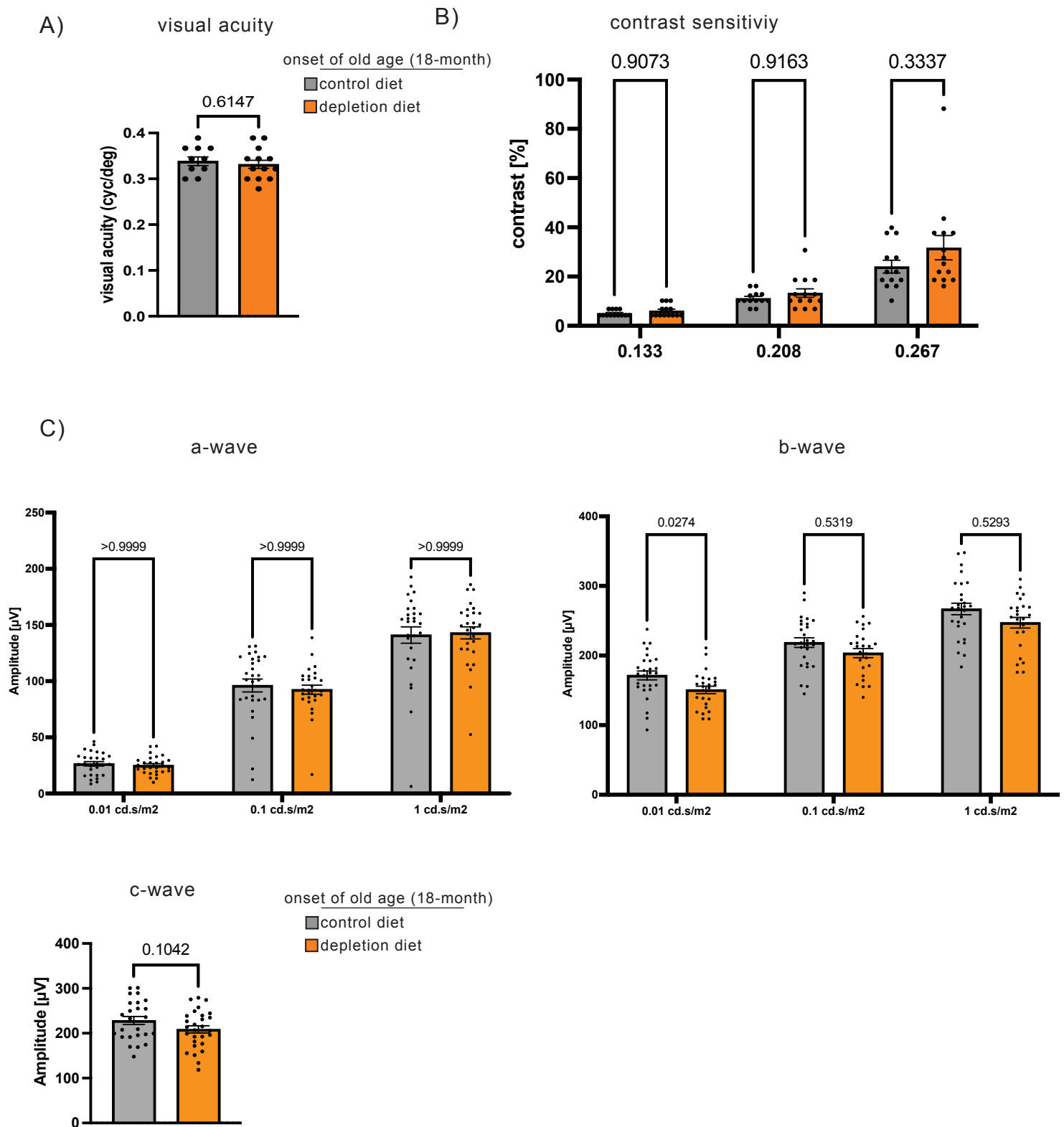

Supplement: Supplementary file 1 — Additional file 1: Supplemental Fig. 1. Microglia depletion in 18 months mice but does not affect visual function. Microglia-depleted middle-aged mice (18-months) showed no difference in (A) visual acuity, (B) contrast sensitivity, and (C) ERG (a, b and c – waves). [file 12979_2023_358_MOESM1_ESM.pdf]
